# Editorial: Preventive and therapeutic potential of physical exercise in neurodegenerative diseases

**DOI:** 10.3389/fphys.2024.1452585

**Published:** 2024-07-08

**Authors:** Xiaocao Liu, Luodan Yang

**Affiliations:** School of Physical Education and Sports Science, South China Normal University, Guangzhou, China

**Keywords:** exercise, prevention, therapeutic potential, neurodegeneration, neurodegenerative diseases

## Introduction

Neurodegenerative diseases, such as Alzheimer’s disease (AD), Parkinson’s disease (PD), and multiple sclerosis (MS), are characterized by the progressive loss of neuronal structure and function, leading to debilitating symptoms and diminished quality of life. Traditional therapeutic approaches have predominantly focused on pharmacological interventions, which, while beneficial, often come with limitations and side effects. Consequently, there is growing interest in alternative and complementary therapies, with physical exercise emerging as a promising candidate. The primary objective of this Research Topic is to deepen our knowledge of the role of physical exercise in neurodegenerative diseases. We aim to contribute by highlighting the mechanisms through which exercise exerts its neuroprotective effects, the most beneficial types of exercise, and the practical implications for clinical practice and public health. This Research Topic includes four publications: two study protocols, one original research article, and one review article. These articles provide new insights and practical strategies for using physical activity and exercise training in the prevention and treatment of neurodegenerative diseases. Moreover, they emphasize the integrated regulation of lifestyle, exercise, skeletal muscle, and sensorimotor functions. More importantly, they provide a systemic view of the complex molecular mechanisms characterizing the role of exercise in reversing neurodegenerative conditions, thereby advancing translational applications in exercise regimen prescriptions for neurodegenerative conditions.

## Integrated refinement of exercise prescription

Physical exercise prescriptions regularly occur at the early stage of neurodegenerative diseases or in high-risk populations to leverage physiological status, maximize exercise availability, support neuroplasticity, and enhance overall wellbeing. Nevertheless, the physical exercise intervention alone is insufficient to respond to the complexity of neurodegenerative diseases, such as multiple sclerosis (MS), which are characterized by disturbances in skeletal muscle, neuromuscular, and cognitive functions. With this regard, Arntzen et al. demonstrated that multidisciplinary guidance, incorporating physical, neurological, and social engagement (e.g., employment support), enhances the efficacy of exercise interventions in MS patients with mild disabilities. Tailoring exercise programs to the target population, considering cultural or regional preferences, and using effective intensities to induce neuroplasticity are crucial to increasing patient participation and intervention efficacy in AD. This approach, as highlighted by Gluck et al., enhances the robustness of personalized physical exercise interventions for reducing AD risk for aged African Americans. Overall, when prescribing exercise regimens for high-risk or early-stage neurodegenerative disease populations, it is essential to incorporate multidisciplinary interventions and tailor the programs to cultural relevance to ensure efficacy.

## Broader context and implications

As an external stimulus, the systemic physiological, biological, and neurological modulation induced by physical exercise intervention, as well as the relevant mechanisms of action, underscores the importance of developing optimal comprehensive exercise strategies. These strategies should include optimal exercise regimens, personalized interventions to account for individual and disease-specific differences, and considerations for long-term adherence and safety. Apart from brain and recognition-mediated modulation driven by physical exercises, Eijgen et al. found that high-intensity exercise serves as a supplementary treatment for retinal nerve degeneration in glaucoma. By targeting both ocular and systemic vascular functions, this study highlights the multifaced benefits of exercise in managing neurodegenerative disease progression. Recently, defective insulin signaling in the brain has been closely linked to neurodegenerative diseases, and emerging experimental results indicate defective insulin regulation functions as a major molecular denominator contributing to neuronal dysfunction. Mechanically, the role of insulin regulation in the benefits of exercise on the nervous system has been systematically summarized by Shen et al. An intricate network has been addressed linking exercise-associated insulin modulation in neurodegenerative disease with metabolic factors, including mitochondrial dysfunction, inflammation, excessive oxidative stress, autophagy defects, and Endoplasmic reticulum stress. Despite research limitations, identifying impaired metabolic parameters as potential mediators of the insulin signaling-neurodegeneration axis regulated by exercises may lead to the development of effective exercise prescriptions for this devastating disease.

## Epilogue

Growing evidence supports a compelling clinical and epidemiological association between physical activity and neurodegenerative diseases. Apart from genetic predispositions, populations with unhealthy lifestyles (sedentary and physically inactive) have a significantly higher risk of developing neurodegenerative disorders, and *vice versa*. To date, ongoing efforts have been made to characterize the role, function, and mechanisms of exercise as a clinical prescription for the prevention, alleviation, and management of neurodegenerative diseases. Consequently, refining exercise regimens that consider the efficacy and effectiveness specific to each neurodegenerative disease is crucial for advancing therapeutic applications. For this purpose, the parameters of exercise (frequency, intensity, volume, and type) and their relationship (reciprocal, independent, dispensable, predisposing) with metabolic signaling are crucial to maximizing the nervous system and overall health outcomes in neurodegenerative conditions. By adhering to these concepts and integrating the findings from this collection with the available literature on the topic, we hope to inspire further research and clinical applications that use physical exercise to improve the health of individuals with neurodegenerative diseases.

